# Understanding the role of emotion and expertise in psychotherapy: An application of dynamical systems mathematical modeling to an entire course of therapy

**DOI:** 10.3389/fpsyt.2023.980739

**Published:** 2023-04-11

**Authors:** Patricia Diaz, Paul R. Peluso, Robert Freund, Andrew Z. Baker, Gabriel Peña

**Affiliations:** ^1^Department of Clinical Mental Health Counseling, Bellevue University, Bellevue, NE, United States; ^2^Department of Counselor Education, Florida Atlantic University, Boca Raton, FL, United States; ^3^Department of Counseling, Lynn University, Boca Raton, FL, United States; ^4^Comprehensive Wellness Center, Lantana, FL, United States

**Keywords:** therapeutic relationship, master therapists, dynamical system (DS), psychotherapy, affect coding, emotion

## Abstract

**Introduction:**

The therapeutic relationship continues to be one of the most important factors in therapeutic outcomes. Given the place of emotion in the definition of the therapeutic relationship, as well as the demonstrated positive impact that emotional expression has on therapeutic process and outcome, it stands to reason that studying the emotional exchange between the therapist and client further would be warranted.

**Methods:**

This study used a validated observational coding system--the Specific Affect Coding System (SPAFF) and a theoretical mathematical model to analyze behaviors which make up the therapeutic relationship. Specifically, the researchers used to codify relationship-building behaviors between an expert therapist and his client over the course of six sessions. Dynamical systems mathematical modeling was also employed to create “phase space portraits” depicting the relational dynamics between the master therapist and his client over six sessions.

**Results:**

Statistical analysis was used to compare SPAFF codes and model parameters between the expert therapist and his client. The expert therapist showed stability in affect codes over six sessions while the client’s affect codes appeared to be more flexible over time, though model parameters remained stable across the six sessions. Finally, phase space portraits depicted the evolution of the affective dynamics between the master therapist and his client as the relationship matured.

**Discussion:**

The clinician’s ability to stay emotionally positive and relatively stable across the six sessions (relative to the client) was noteworthy. It formed the basis for a stable base from which she could explore alternative methods to relate to others that she had allowed to dictate her actions, which is in keeping with previous research on the role of therapist facilitation of the therapeutic relationship, emotional expression within the therapeutic relationship, and influence of these on client outcomes. These results provide a valuable foundation for future research on emotional expression as a key component of the therapeutic relationship in psychotherapy.

## Introduction

The therapeutic relationship continues to be one of the most important factors in therapeutic outcomes. Defined by the APA’s Third Interdivisional Task Force on Evidence Based Relationships and Responsiveness, as “(t)he *feelings* and attitudes that therapist and client have toward one another, *and the manner in which these are expressed*” [(1), p. 3, *italics added for emphasis*], the emphasis on emotional expression is central to the definition of the therapeutic relationship. The therapeutic relationship is not unlike social relationships, though it is unique, and considered to be “therapeutic in and of itself” [(2), p. 56]. In fact, the task force concluded that effective therapeutic relationships make “substantial and consistent” contributions to therapeutic outcomes, and facilitate improvement regardless of the type of therapy conducted [(3), p. 329]. According to Norcross and Lambert (1), the therapy relationship accounts for 15% of the explained variance in psychotherapy outcome attributable to therapeutic factors. This was the third largest percentage, following unexplained variance (35%), and patient contribution (30%), but ahead of treatment method (10%) and the individual therapist (7%). Taken together, the individual therapist and therapeutic relationship make up 22% of the outcome variance. This led Norcross and Lambert to their conclusion that “the *person* of the psychotherapist is inextricably intertwined with the outcome of psychotherapy” (2019, p. 7).

## Research on emotional expression as a relationship element

The same APA task force commissioned a series of meta analyses to investigate different aspects of the therapeutic relationship. Fluckiger, Del Re, Wampold, and Horvath (4), conducted several meta analyses that replicated previous findings about the relationship of the therapeutic alliance (one aspect of the therapeutic relationship) to patient outcome (*r* = 0.28). However, they also found that there were no differences in effect size between alliance and outcome for studies that were random-controlled trials and other research designs. They also noted specifically that the therapeutic alliance is dependent on “creating a *warm emotional bond* or collaborative attachment with the patient” (p. 61, *italics added*). Peluso and Freund (5) contributed to the task force by conducting a series of meta analyses that examined the impact of emotional expression in therapy on the therapeutic relationship and on therapy outcomes. They found significant medium effect sizes for emotional expression and the therapeutic process for clients (*d* = 0.63) and for therapists (*d* = 0.54). In terms of the relationship to outcomes, a medium effect size for therapist emotional expression and therapeutic outcome was observed (*d* = 0.56), with a medium to large effect size for client emotional expression and therapeutic outcome (*d* = 0.85). Given the place of emotion in the definition of the therapeutic relationship, as well as the demonstrated positive impact that emotional expression has on therapeutic process and outcome, it stands to reason that studying the emotional exchange between the therapist and client further would be warranted. Currently, however, there is a paucity of research on how these therapists mobilize emotional and relationship-building behaviors to effect positive change in their clients, which has led researchers to call for further investigation into this factor (5–11).

## Use of dynamical systems to assess relationship dynamics

Norcross and Wampold (3) in their summary of the APA task force recommended that future research include an investigation of relationship components on a second-by-second basis, as well as the use of designs that investigate more complex interactions. They also suggested that researchers use observational methods, and attempt to understand the therapist contribution to the therapeutic relationship. Dynamical systems (DS) is an approach that has been used to measure complex phenomena, both mechanical and human, like relationships that can change over time (12). Simply put, “DS is the measure of changes over time using mathematical equations” [(6), p. 224], which makes it a powerful tool for investigating powerfully dynamic interactions, like the therapeutic relationship or other aspects of psychotherapy (13, 14). In fact, Baker et al. outline that there are two aspects of investigation with DS, time-span (ranging from second-to-second, to session-to-session, and beyond), and the element of psychotherapy being studied (e.g., emotional exchanges, word choices, and coordination of movement). Following their investigation, Peluso and Freund (5) recommended that DS modeling could “provide a rich graphical description of the dynamics of the relationship to therapists and researchers alike” (p. 449). Tschacher and Haken (15) described how DS approaches were uniquely suited to psychotherapy research as it can successfully temporal aspects of therapy, as well as the deterministic features (i.e., attractors), and stochastic (changing) elements within the dyad. While it may be a potentially useful method for studying the dynamics and processes within the therapeutic relationship, “these methods have rarely been used in psychotherapy research” [(16), pp. 607].

Perhaps one of the most successful applications of DS modeling to the effects of emotional expression on a relationship is the work of John Gottman and his associates. Using an observational coding system, physiological measures, and DS modeling, he was able to predict, with approximately 94% accuracy, which romantic relationships would end in divorce or remain together 5 years later (17–19). Liebovitch et al. (20) modified Gottman’s original dynamical systems nonlinear equations [equations are described below, see (18) for more details] to apply them to therapists and clients. Peluso et al. (21) then used these equations to create simulations of different relationship dynamics by manipulating the parameters of the mathematical model this work revealed that the internal dynamics of the relationship could be meaningfully modeled. Luedke et al. (22), following from recommendation of Peluso et al. (18), implemented a process-based observational method of analyzing the therapeutic relationship, using a modified version of Coan and Gottman’s (17) Specific Affect Coding System (or, SPAFF). The researchers cataloged the second-by-second affective exchanges of clinicians and clients, and successfully predicted client retention in recorded sessions of psychotherapy (22).

Over the last few years, other researchers have successfully used nonlinear equations of Peluso et al. (18) to model aspects of the therapeutic relationship or therapeutic process. Soma et al. (23) used dynamical systems analysis to analyze the degree to which fluctuations in the fundamental frequency of either the therapist’s or client’s voice (as a measure of arousal) would influence the other person. Though they did not use an ordinary differential equation (ODE), to model the dynamics (as Peluso et al. (21) did) their findings did show that, using a dynamical systems framework, a mutual association between therapist and client does exist. Li et al. (24) and Li and Kivlighan (16) also applied a version of equations of Peluso et al. (18) to ascertain the dynamics of the relationship between therapist and client using ratings of the working alliance as a measure of therapist responsiveness, and the influence and of each person on the other on clinical outcomes. They concluded that this approach was “a viable technique in modeling nonlinear dynamic therapy processes under other theoretical frameworks in future research” [(24), p. 12].

Most recently, Baker et al. (6) used Peluso et al. (18) DS modeling and the same affect coding system (SPAFF) following Luedke et al.’s (22) method and applied it to actual therapy sessions from separate theoretical orientations (cognitive-behavioral therapy, emotion-focused therapy, and psychodynamic therapy) conducted by three expert therapists from each approach (Judith Beck, Leslie Greenberg, and Nancy McWilliams) who saw the same two clients. They found that: (1). DS mathematical modeling could be used to accurately capture and explore the emotional exchanges of the therapeutic relationship and (2) expert therapists, despite using vastly different theoretical approaches, construct very similar relationship dynamics that are dependent on the client rather than school of therapy. Specifically, they found that each of the three therapists had similarities in the graphic representation of the relationship models for the female client and for the male client, and less similarity within each therapist. This provided evidence for the tailoring or relationships based on the client, as well as evidence for the relationship as a common factor rather than a specific ingredient based in a theoretical approach (1, 2, 6). In their conclusion, they recommended following up their findings by applying DS mathematical modeling to a full course of therapy, and consider how expert (or “master”) therapists develop the therapeutic relationship over time.

## Expert psychotherapists, *Psychotherapy over time* video series and *How master therapists work*

Some clinicians can form effective therapeutic relationships better than others. These differences in ability to form these relationships have been shown to impact clinical effectiveness (2). “Master” or expert therapists differ from novices, and even professional therapists, in several important ways (8, 25–27). A hallmark of these experts is the clinician’s technical skill, as well as her response to larger contextual issues (7, 8). In order to demonstrate this competency, one must have exceptional relationship skills, and cultivate strong working alliances (8, 28). Several studies and simulations have demonstrated that high-performing therapists have significantly better outcomes than others. For example, Nissen-Lie et al. (10) differentiated therapists based on over 6,000 client outcomes. Specifically, they found that the majority of therapists (approximately two-thirds) could not be categorized because their clinical outcomes were mixed (some good, some poor). However, approximately 15–20% of clinicians could be identified with consistently good outcomes, and approximately the same level for clinicians with consistently poorer outcomes (11). More recently, Pereira et al. (25) conducted a systemic review of highly effective therapists and found that emotional expression was a significant predictor of therapist effectiveness. It stands to reason that clinicians who consistently have better outcomes, must construct their therapeutic relationships differently than other professional clinicians. In fact, their analysis lead Peluso and Freund to speculate that “there might be an element of mediation between how experience and mastery affect the expression of affect and its impact on clinical outcomes” (2019, p. 448). Unfortunately, there is a lack of definitive research on how expert therapists accomplish this, let alone utilize an effective relationship to effect change in their clients (7, 8, 25–27, 29).

In 2006, the APA created a video series, *Psychotherapy Over Time* as a way to observe acknowledged experts in the field of psychotherapy working with a client. This series went beyond a “one-shot” demonstration, which is prevalent in many therapy training videos. The first video in the series was by series creator, Dr. Jon Carlson, who demonstrated Adlerian theory (41). What was compelling about Carlson’s series was that approximately 10 years after the videos were recorded, Sperry and Carlson (26) published the book *How Master Therapists Work: Effecting Change from the First through the Last Session and Beyond*. In their text, Sperry and Carlson expand on the specific characteristics of master therapists, thusly:

They are voracious learners; draw extensively from accumulated experience; value cognitive complexity; *are emotionally receptive and non-defensive*; are mentally healthy and mature individuals who *attend to their own emotional well-being*; are aware of *how their emotional health affects work* quality; *possess strong relationship skills and are experts in using those skills in therapy*; trust their clients; are culturally competent; and *believe that the foundation for therapeutic change is a strong therapeutic alliance* (p. 16, *italics added for emphasis*).

In fact, this is not very different from the criteria that Hill et al. (8) used to define “expert” therapists: “*the manifestation of the highest levels of ability, skill, professional competence, and effectiveness”* (p. 9, *italics in the original*). In addition, Hill et al. stated that *better* research on expertise in therapy is needed that goes beyond cross-sectional designs that compare novice therapists to experts over longer periods of time.

What is noteworthy about the Carlson videos and book is that their text is an in-depth study of Carlson’s original videos, which includes both Carlson’s reflections on the sessions, as well as the reflections of the client who participated (which we will utilize in our analysis) nearly a decade later. Given Baker et al.’s (6) and Hill et al.’s (8) recommendation above, and with this definition of mastery fitting with the interest in investigating the emotional expressions in the therapeutic relationship, we felt that both the six-session videos, as well as the reflection contained in the subsequent book was a unique combination of works that add an enriched analysis to the mathematical modeling of the relationship between Carlson and his client.

## The present study

There remain questions about what aspects of the therapeutic relationship are stable and which ones change over the course of therapy (1–3). Peluso and Freund (5) speculated that emotional expression would be an aspect of the therapeutic relationship that would change from session to session but did not have any conclusive evidence of exactly how. In addition, there was little empirical evidence of the degree to which client therapist emotional expression changes from session to session and how this impacts the overall therapeutic relationship, and ultimately therapeutic outcome. One of the limitations of work of Baker et al. (6) was that each expert only had one session with either of the clients on their video, rather than multiple sessions with the same therapist. The present study is an extension of this work and includes an in-depth investigation into the evolution and change of the therapeutic relationship within a single, brief course of therapy, conducted by an expert therapist, that goes from the establishment of the relationship through the working phase and ultimately to termination. Given that most courses of psychotherapy are brief (less than eight sessions), this is a reasonable analog of a successful therapy as defined—at the time of termination and retrospectively—by both the therapist and the client (2, 26). DS modeling of the emotional expressions across multiple sessions of therapy will provide a measure of the changes in emotional expression within a session, as well as between sessions across the entire course of this one case of therapy. The present study builds on the foundations of research established by Peluso and his colleagues, as well as others, to use DS modeling in order to address the questions resulting from previous studies on the therapeutic relationship. The focus of this paper will be on the dynamics of the relationship, particularly the emotional expression of the therapist and client, and whether and how they change. This will be investigated through the following research questions:

*Research Question 1*: How does emotional expression for both the therapist and client change across the entire course of therapy (as measured by SPAFF observational codes). Given the lack of consensus on the subject, this hypothesis is non-directional.

*Research Question 2*: How do the emotional dynamics of the relationship for both the therapist and client change across the entire course of therapy (as measured by the DS mathematical model parameters of initial state, inertia, uninfluenced steady state, and influence function thresholds). Again, given the lack of consensus on the subject, this hypothesis is non-directional.

*Research Question 3*: How does the overall therapeutic relationship change across the entire course of therapy? As this will be depicted by phase-portraits that will graphically represent the mathematical models across the six sessions, this will be a qualitative analysis of each of the portraits to examine how each session is similar or differs from one another [similar to analysis of Baker et al. (6)].

## Methods

### Participants

The APA-produced six DVD series *Psychotherapy Over Time* featured Dr. Jon Carlson, and his client, Aimee (41), which was used for the coding and mathematical modeling. Permission to use the APA published *Psychotherapy Over Time* series was obtained from The American Psychological Association for research purposes (G. VandenBos, personal communication, June 13, 2014).

#### Therapist

Dr. Jon Carlson (1945–2017) was a highly established, peer-nominated expert therapist and was well-regarded by his peers in the disciplines of counseling, psychology, and medicine. Carlson earned doctoral degrees in counseling and clinical psychology and emphasized the use of Adlerian or Individuals Psychology in his work with clients (26). He received lifetime contributions awards from several professional organizations, including the American Psychological Association and the American Counseling Association, and was named one of the Living Legends in Counseling in 2004. He published 60 books, 170 professional articles, and has produced over 300 video training programs (Lake Geneva Wellness Clinic, n.d., Sperry & Carlson). Using criteria of Hill et al. (8) for evaluating expertise, the researchers were comfortable accepting Carlson as an identified expert therapist whose skills merited closer examination.

#### Client

At the time of her sessions with Carlson, Aimee was a 30-year-old, single mother to two boys, ages 12 and 13. According to Sperry and Carlson (26), Aimee was employed as a limousine driver while attending graduate school part-time and reportedly sought counseling in order to better cope with resentment toward her mother. Prior to counseling, Aimee endorsed taking a passive and avoidant approach in life and, in particular, with her mother. Some of her additional concerns included the impending release of her ex-husband from prison, whom she had divorced due to his abusive behaviors. Aimee reported having headaches, poor sleep, anxiety, depression, and nightmares at the time of counseling. She would be most accurately diagnosed with Unspecified Depressive Disorder with Mixed Features and Dependent Personality Disorder with Obsessive Compulsive Traits (26).

### Measures

#### The specific affect coding system^1^

The Specific Affect Coding System, or SPAFF, was originally developed (30) and validated (31) for the examination of the marital relationship. SPAFF has 20 separate behavioral codes (17). It includes one affect code for neutral behavior, seven positive affect codes (affection, high validation, humor, interest, surprise/joy, low validation, and tense humor), and 12 negative affect codes (contempt, belligerence, criticism, stonewalling, defensiveness, high domineering, low domineering, anger, sadness, whining, disgust, and tension) (32). These codes are applied in real-time *via* marked keys on a computer keyboard while watching split-screened, video-taped interactions uploaded into observational research software. This creates a second-by-second data stream of the interaction.

Specific Affect Coding System was initially used in examining marital conflict interactions (17), although the codes within the system can be applied to any type of conversation in the relationship (18). SPAFF has now been successfully used to assess other types of relationships including: triadic parent-baby interactions (18), the relationship between medical doctors and their patients (33), and most recently, to therapeutic relationship (22, 34, 35). Van Walsum (33) indicated negative emotion had an effect on simulated patients working with student physicians, and Erzar et al. (34) demonstrated SPAFF could be applied to the counseling relationship using a Slovenian translation of the system. Luedke et al. (22) examined interactions between therapists and their clients using SPAFF and found significant differences in the affective behavior of clients who returned to therapy and those who dropped out of therapy.

### Procedure

Once recorded, filmed sessions were edited down into three, 15-min segments to maintain fidelity to procedure of Coan and Gottman (17) to prevent coder fatigue. Each 15-min segment requires 45 min of coder time (22). While not all tapes could be evenly edited into 15 min segments, the average length of sessions recorded was 45 min. Furthermore, sessions that were longer or shorter than 45 min tended to vary from, on average, by less than 1 min. As a result, for all data analyses, percentage of time was used to account for any discrepancies.

#### Video affect coding procedure

The therapist’s and client’s affective exchanges were coded using the 20-code SPAFF system (32). The manual provided by the Gottman Institute (32) was the basis for training the coders employed by the current study. All coders employed in the current study were involved in intensive three-day (8 h per day) SPAFF coding training conducted by a Gottman Institute coding trainer. The coders then coded 10 videos over the course of the next few weeks, and maintained inter-rater reliability with the coding trainer (gold standard coder) for the last six consecutive videos, as per the training procedure established by the Gottman Institute. Coders are considered to have achieved inter-rater reliability in SPAFF coding if their codes show windowed kappa, free-marginal kappa, and windowed free-marginal kappa coefficients of 0.6 or higher, which is slightly lower than is expected, due to SPAFF being a real-time coding system with 21 codes that is used over a longer period of time compared to other observational coding systems (J. Gottman, personal communication, February, 1, 2016).

The coding procedure required three viewings of each 15-min session segment. The first viewing is to establish a contextual baseline, the second viewing is to code one of the parties (i.e., the therapist), and the third viewing is to code the other party (i.e., the client). The coded data is then exported for manipulation in statistical programs. Twenty-five percent of coded videos were “double coded” by two independent coders and a kappa over 0.6 must be obtained in order to ensure continued inter-rater reliability (coders for this project demonstrated kappas over 0.7). This procedure was established in Gottman’s research during the validation of SPAFF (17), and has been used in research on the therapeutic relationship (22, 35).^2^

#### Data preparation for modeling

Following Gottman’s research protocol, using Noldus Observer v. 11, each second of the session was assigned a code^3^ and each code was weighted; then every 6 s of material was summed to create 150 data points from 900 s (15 min) of video (18). The weighting of the codes varies from −4 to +4 so a range of −24 to +24 for each unit is possible (for original weighting see, (18)). Luedke et al. (22) posited that the therapeutic relationship carries an inherent power differential that is not assumed in a marital relationship, and completed a discriminant function analysis on a sample of SPAFF-coded therapy segments. Using the structure matrix coefficients derived from the discriminant function analysis as a numerical basis, Peluso (36) created a new weighting system with separate weights for clients and therapists, which was employed in the current study.

#### Dynamical systems mathematical modeling

We will briefly describe the DS equations initially developed from the work of Gottman and his colleagues (18, 19) and modified by Liebovitch et al. (20) and Peluso et al. (21).^4^ As mentioned above, DS equations assess changes over time, as a result, they are often expressed as differential equations, where there is a change in a measure (*dT, dC*), over a period of time (*dt*), and expressed as a mathematical function. In our case, the equations that we used are below (see Equation 1).


$$\frac{dT}{dt}m_{1}T+b_{1}+c_{1}F_{c}\left(C\right)$$

(1) $$\frac{dC}{dt}=m_{2}C+b_{2}+c_{2}F_{T}\left(T\right)dt$$

Each variable in the equation represents either a score at a moment in time, or a parameter that is a mathematical representation of an element of the phenomenon being studied (in this case, the therapeutic relationship). In Equation 1, *b_1_* and *b_2_* are the initial state for the therapist and the client, and *m_1_* and *m_2_* are each person’s inertia (or their tendency to stay in a previous emotional state). According to Baker et al. (6):

These four parameters (called the *uninfluenced parameters*^5^) are derived using a least squares method, and computed by summing the scores of one partner when the other person is neutral, and compared the changes in scores for each of these at moment *t* + 1. The *initial state* parameter is derived by total positive and negative scores, when the other person’s score is zero (or is having no influence). Broadly speaking, this can be thought of as the individual’s unique disposition (positive, negative or neutral), that introduces a constant… via the *b*_1_ and *b*_2_ parameters. The *inertia* parameter is “the tendency of remaining in the same state for a period of time” (19, p. 114), and is estimated by taking an average of positive scores minus negative scores when the other partner’s score was zero^6^. The greater a person’s inertia is, the less likely they are to be open to influence from the other person (pp. 225-226).

The next parameters, *c_1_F_T_(T)*, *c_2_F_C_(C)* are the influence functions of the therapist on the client, and of the client on the therapist, respectively (6).

We generated the initial state, inertia, uninfluenced steady state^7^, the thresholds for the influence functions in the negative and positive regimes, as well as the strength and threshold for the repair parameter using procedure Gottman et al. (18) for deriving parameters used in the DS equations.^8^ This provided the necessary parameters to create unique mathematical models for each of the six therapy sessions for both therapists and clients, in accordance with approach of Peluso et al. (21). The key difference in this analysis is that each of the parameters were derived from the weighted and summed SPAFF data coded in each of the six sessions.

Using an ODE solver in MATLAB^9^ [again, see (6, 20), for details] to show the changes over time, the phase-space portrait was created based on solutions to the two ordinary differential equations in Equation 1. An advantage of using differential equations is that they allow for continuous (rather than discrete) analysis of the dynamical system and create a more realistic interaction between the two partners, by creating trajectories from *each potential starting point* (6, 12).^10^ This approach allows for a more complete exploration of the system, which will be illustrated below.

#### Data analysis

In order to explore research questions 1 and 2, a Kolmogorov–Smirnov Exact test will be used on the percent of time spent in each SPAFF code for both the therapist and the client. This is a non-parametric test that is appropriate for several reasons. First, there were fewer than 30 observations per variable (in fact, there were exactly six), and second, the scores themselves were numerically less than 5, which made a chi-square (the usual method for investigating) invalid. Secondly, we chose to compare scores to each other, rather than impose a normal distribution to the scores, as we wanted to see if they differed significantly from one another, from session to session. In instances like the present study, the Kolmogorov–Smirnov Exact test is recommended (37).

Research question 2 will also be answered using a Kolmogorov–Smirnov Exact test, using the parameters for the DS models (initial state, inertia, positive and negative influence thresholds, repair strength, and repair location) for the therapist and client for all six sessions. Furthermore, the DS models will be graphically depicted using phase portraits which will allow for a visual inspection of the relationship dynamics as modeled (6). These will be used specifically to respond to research question 3.

## Results

We will begin with an analysis of the individual SPAFF codes for both Jon Carlson and Aimee that were detected across all six sessions. Next, we will present an analysis of the mathematical models of all six sessions, beginning with the model parameters that were derived from the SPAFF data, and then we will evaluate the phase-portraits for the overall dynamics of the relationship at each session. Where applicable, all alpha levels were set a 0.05.

### Comparison of affect codes

One of the overarching questions posed in this paper is whether there were any systemic differences in the therapeutic relationship, between Jon Carlson and Aimee as indicated by SPAFF coding of the affect across the sessions. Table 1 lists the number of seconds and the percentage of Carlson’s individual SPAFF codes, as well as the total positive and total negative codes. The number of seconds and percentages of SPAFF codes were compared across the six sessions using a Kolmogorov–Smirnov Exact test.

**Table 1:** SPAFF codes for Dr. Jon Carlson over six sessions.

Code	Session 1	Session 2	Session 3	Session 4	Session 5	Session 6
Low domineering	13 (0.50)	18 (0.67)	112 (4.15)	47 (1.85)	62 (2.30)	46 (1.70)
Tension	8 (0.30)	6 (0.22)	74 (2.74)	31 (1.22)	63 (2.33)	40 (1.48)
Tense humor	5 (0.19)	1 (0.04)	6 (0.22)	1 (0.04)	9 (0.33)	12 (0.44)
Neutral	2,132 (81.19)	2047 (75.81)	2,128 (78.81)	1951 (76.87)	1906 (70.59)	1859 (68.85)
Interest	130 (4.95)	79 (2.93)	136 (5.04)	150 (5.91)	142 (5.26)	103 (3.81)
Low validation	194 (7.39)	252 (9.33)	118 (4.37)	226 (8.90)	240 (8.89)	212 (7.85)
High validation	135 (5.14)	289 (10.70)	119 (4.41)	126 (8.90)	263 (9.74)	405 (15.0)
Affection	8 (0.30)	8 (0.30)	3 (0.11)	0	8 (0.30)	13 (0.48)
Humor	0	0	0	4 (0.16)	7 (0.26)	7 (0.26)
Surprise/Joy	1 (0.04)	0	4 (0.15)	2 (0.08)	0	3 (0.11)
Total positive	468 (17.82)	628 (23.26)	380 (14.07)	508 (20.02)	660 (24.44)	743 (27.52)
Total negative	26 (0.99)	25 (0.93)	192 (7.11)	79 (3.11)	134 (4.96)	98 (3.63)

Number of Seconds per code (Percentage in parentheses).

Looking at Table 1, the SPAFF codes that were detected for Carlson included: Low Domineering, Tension, Tense Humor, Neutral, Interest, Low Validation, High Validation, Affection, Humor, and Surprise/Joy. In addition, we computed the Total Positive and Total Negative scores. The only SPAFF code that was significantly different from session to session was Humor, *D*(5) = 0.500, *p* < 0.05. If we look at Table 1, Carlson did not have any seconds of humor in sessions 1–3, but did in sessions 4–6, though the length was fewer than 10 s in each instance. What may be more interesting is the fact that none of the other SPAFF codes differed significantly from session-to-session, including the Total Positive and Total Negative scores.

An analysis of the SPAFF codes for Aimee over the span of the six sessions was also conducted. Table 2 lists Aimee’s individual SPAFF codes included: Disgust. Low Domineering, Tension, Tense Humor, Sadness, Neutral, Interest, Low Validation, High Validation, Affection, Humor, and Surprise/Joy. In addition, just as with Carlson, we computed both the number of seconds, and the percentages as well as the Total Positive and Total Negative scores. Unlike Carlson’s scores, Aimee’s scores did show significant differences from session to session for the following SPAFF codes: Disgust [*D*(5) = 0.833, *p* < 0.05], Low Domineering [*D*(5) = 0.833, *p* < 0.05], Interest [*D*(5) = 0.711, *p* < 0.05], Affection [*D*(5) = 0.833, *p* < 0.05], Humor [*D*(5) = 0.667, *p* < 0.05], and Surprise/Joy [*D*(5) = 0.667, *p* < 0.05]. Neither Total Positive or Total Negative were significantly different, however. Looking at Table 2, Disgust and Low Domineering only appeared in one session out of the five, and even then for less than 10 s. Interest (typified by asking questions), however, showed a marked increase in session 4. Like the finding for Carlson, Humor did increase in sessions 5 and 6, while Surprise/Joy was present in Sessions 2 and 4 (but not other sessions).

**Table 2:** SPAFF codes for Aimee over six sessions.

Code	Session 1	Session 2	Session 3	Session 4	Session 5	Session 6
Disgust	0	0	8 (0.30)	0	0	0
Low domineering	0	0	0	0	5 (0.19)	0
Tension	277 (10.55)	132 (4.89)	584 (21.63)	168 (6.62)	583 (21.59)	140 (5.19)
Tense humor	5 (0.19)	1 (0.04)	4 (0.15)	0	10 (0.37)	10 (0.37)
Sadness	89 (3.39)	19 (0.70)	0	21 (0.83)	0	0
Neutral	2,176 (82.86)	2,403 (89.00)	1984 (73.48)	2047 (80.65)	2,107 (74.70)	2,279 (84.41)
Interest	2 (0.08)	1 (0.04)	0	54 (2.13)	0	7 (0.26)
Low validation	62 (2.36)	100 (3.70)	120 (4.44)	164 (6.46)	71 (2.63)	189 (7.0)
High validation	15 (0.57)	40 (1.48)	0	79 (3.11)	7 (0.26)	34 (1.26)
Affection	0	0	0	0	3 (0.11)	0
Humor	0	0	0	0	4 (0.15)	4 (0.15)
Surprise/Joy	0	4 (0.15)	0	5 (0.20)	0	0
Total positive	7 (3.01)	145 (5.73)	120 (4.44)	302 (11.90)	81 (3.0)	230 (8.52)
Total negative	371 (14.13)	152 (5.63)	596 (22.07)	189 (7.45)	598 (22.15)	187 (6.93)

Number of seconds per code (Percentage in parentheses).

Taken together, in terms of coded emotional expression in sessions over time for this particular series of therapy sessions, the therapist displayed fewer emotion codes than the client, and the proportion of therapist codes did not significantly change (except one). At the same time, the client did show significant changes in a number of codes over the six sessions. However, in order to assess the dynamic nature of the relationship, and the impact of this on the overall system, we will consider the results of the mathematical modeling next.

### Mathematical modeling of the therapeutic relationships

#### Comparisons of parameters across sessions

Following the procedure laid out by Baker et al. (6) using Peluso et al.’s (2012) equations, mathematical models and parameters for all six sessions were computed. Table 3 lists the derived parameters for all six sessions for Jon Carlson and Table 4 lists the parameters for Aimee. Just as before, in order to determine if the parameters differed from significantly from session-to-session a Kolmogorov–Smirnov Exact test was employed. For both Jon Carlson and Aimee, the parameters of their models were not significantly different from one another, suggesting that despite the differences detected in Aimee’s individual SPAFF codes described above, the model parameters for both Carlson and Aimee are relatively consistent across the sessions over time. One explanation for this is that the parameters are derived primarily from summed scores when one partner or another is negative or positive. In the analyses above, neither Carlson or Aimee’s total positive or total negative scores were significantly different. However, as we will see below, there are dynamic variations in each session that are worth exploring.

**Table 3:** Dynamical systems model parameters for Jon Carlson.

	a2	r2	UnSS	*n*th	*p*th	kr	sr
Session 1	0.5692533	0.3138643	0.82965119	−2.2	−0.5	−2.6	4.1
Session 2	0.7704783	0.3137779	1.1227827	−0.5	0.6	−1	0.4
Session3	0.01937659	0.456303	0.03563858	−3	−1.3	−3.7	1.7
Session 4	0.1355777	0.3912368	0.22271008	−0.6	−0.2	−5.7	1.3
Session 5	0.6156569	0.3420887	0.93577493	−2.7	−1.6	N/A	N/A
Session 6	0.8492836	0.3576016	1.32205124	−0.5	0.9	−1	0.5

a1, initial state; r1, inertia; UnSS, uninfluenced steady state; *n*th, influence function negative threshold; *p*th, influence function positive threshold; kr, threshold of repair; and sr, strength of repair.

**Table 4:** Dynamical systems model parameters for Aimee.

	a2	r2	UnSS	*n*th	*p*th	kr	sr
Session 1	−0.2270265	0.3979142	−0.3770667	−1	1.8	−1.5	1.1
Session 2	0.1203736	0.232378	0.15681364	−1.6	−0.2	−3.7	3.1
Session3	−0.4135209	0.3502402	−0.6364212	−1.2	3.4	−1	1.3
Session 4	−0.1998837	0.1183646	−0.2267192	−2.6	3.4	−1.4	1
Session 5	−0.7585239	0.2088656	−0.9587801	1.4	6.2	N/A	N/A
Session 6	0.1575602	0.3890159	0.25787938	−1.2	0.2	−5	1

a2, initial state; r2, inertia; UnSS, uninfluenced steady state; *n*th, influence function negative threshold; *p*th, influence function positive threshold; kr, threshold of repair; and sr, strength of repair.

#### Phase portrait visualization of the therapeutic relationships

**Figure 1:**
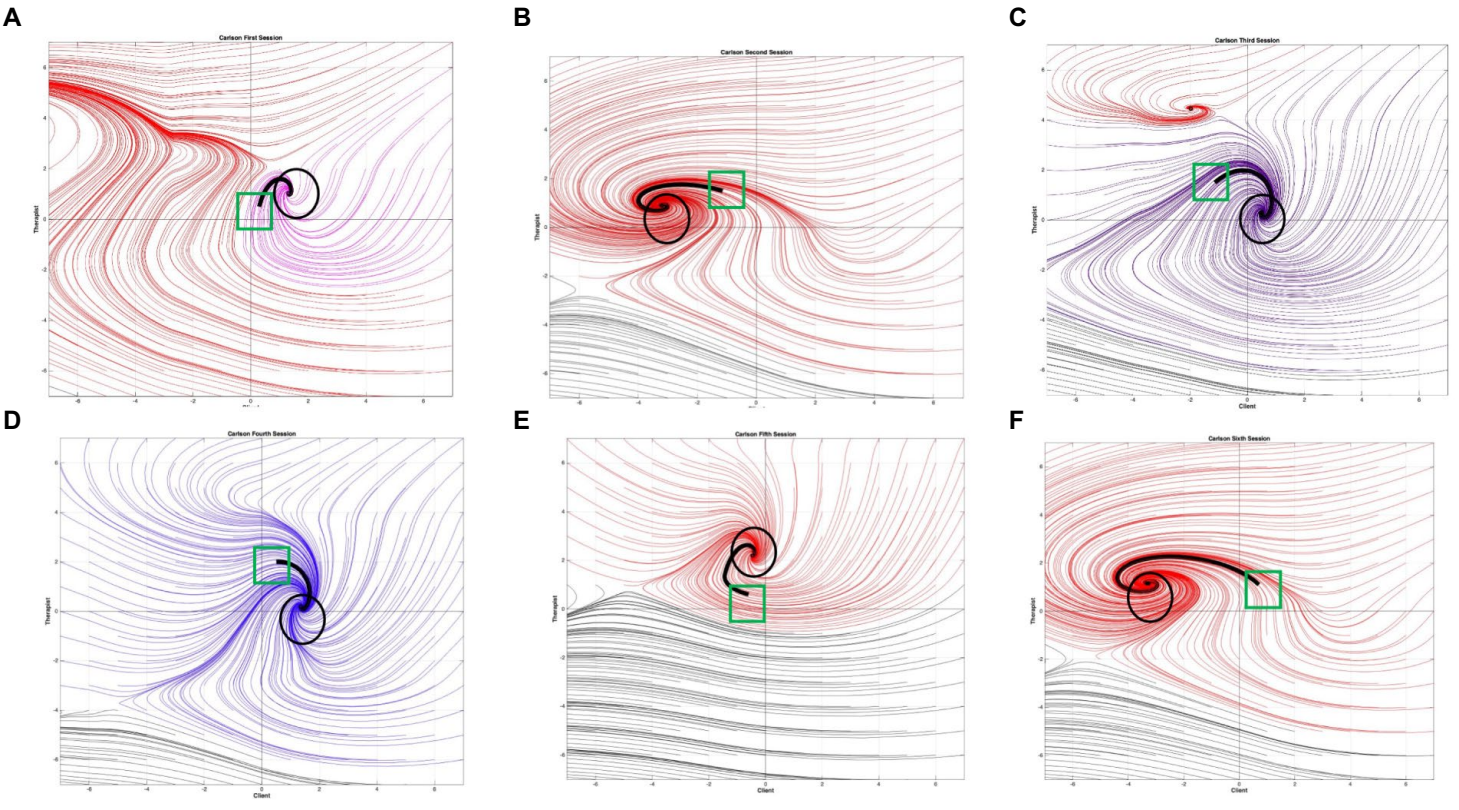
Phase portraits of all six sessions of therapy between Jon Carlson and Aime. **(A)** is the phase portrait for session 1, **(B)** is for session 2, **(C)** is for session 3, etc. Beginning coordinates denoted by green circles, and ending coordinates denoted by black circles. Black line is the trajectory that the session likely took based on the starting coordinates.

The models in the current study were derived from the SPAFF data of the emotional expression in the six sessions of real therapy with Jon Carlson and his client Aimee taken from the *Psychotherapy Over Time* video series (Carlson, 2006). The parameters that are derived from the mathematical models are best considered using a graphic visualization, especially for complex systems (20). The phase-space portraits in Figure 1 shows two-dimensional phase-space portraits of all six sessions,^11^ and were created using the parameters derived from the differential equations (see Equation 1, above listed in Tables 3, 4). This was an iterative process using 10 time-steps, and displays the trajectory lines for every set of all potential starting coordinates for the system, as well as the critical point (s) in the system. Hence, by knowing what the initial starting point is for the session, the actual trajectory of the session can be estimated. This is accomplished in each of the six sessions in Figure 1 by averaging the first 10 percent of the SPAFF codes for therapist and client. In each of the phase portraits in Figure 1 these coordinates (client starting point value on the *x*-axis, therapist starting point value on the *y*-axis) is denoted by a green square. The estimated trajectory of the actual sessions are then drawn on the phase-space portrait (as a black line) to indicate the best estimation of how the math model predicted the quality and endpoint of the relationship in the session where the parameters for the whole system were derived. The quadrant in which the critical points are located is an indication of the quality of the relationship (e.g., a positive–positive quadrant vs. negative–negative quadrant), while the black trajectory line represents the estimated actual endpoint of the therapeutic relationship (6, 18–21). The endpoint coordinates of the trajectory in each of the phase portraits in Figure 1 is denoted by a black circle.

There are several noteworthy aspects of the phase-space portraits for the six sessions as in Figure 1. In the first, third and fourth session (Figures 1A,C,D, respectively), there are attractors in the positive–positive quadrant, with all three having actual trajectories (representing what actually happened, compared to what could have happened) in that quadrant (again, as indicated by the black square). The other three sessions (Figures 1B,E,F) all have dominant attractor points in the therapist positive/client negative quadrant (though it can be argued that in session 5 (Figure 1C), the attractor point is just barely outside of the positive–positive quadrant). While seemingly not as satisfactory as an attractor in the positive–positive quadrant, it is noteworthy that in those sessions, the location of the attractor represents the optimal outcome of the particular session, and that the trajectory from the actual session did move toward the optimal attractor. It is also noteworthy that none of the sessions ended up in the negative–negative quadrant of the phase portrait. This quadrant is indicative of a poor session outcome, with each participant displaying overt negativity, with no attraction to a positive emotional state (6).

## Discussion

The present study used an affect coding system and a dynamical systems approach to mathematical modeling to map the therapeutic relationship of an expert psychotherapist, Jon Carlson and his client Aimee over the course of six sessions in order to illustrate elements of emotional expression and relationship building behaviors by a master therapist and their impact on the client over time. The overarching research questions that were investigated were:

1. How does emotional expression for both the therapist and client change across the entire course of therapy (as measured by SPAFF observational codes)?
2. How do the emotional dynamics of the relationship for both the therapist and client change across the entire course of therapy (as measured by the DS mathematical model parameters of initial state, inertia, uninfluenced steady state, and influence function thresholds)?
3. How does the overall therapeutic relationship change across the entire course of therapy?

These questions followed from previous work and the results of the present study provided some evidence that these dynamics have both elements of stability and change within them. These elements will be discussed below, as well as limitations of the current study and next steps in this line of research.

### Changes in emotional expression between sessions

One of the interesting findings was the consistency of the therapist’s SPAFF codes across the six sessions. At the same time, the ratio of positive to negative scores were as high as approximately 20:1 (session 2) and went as low as 2:1 (session 3), and averaged 10:1. This finding is in line with work from Wolf et al. (27) who found that highly effective therapists displayed less negative affect, and depiction of the stability of therapist demeanor of Wampold et al. (11) in the face of client affect. This is also reflected in Aimee’s scores, which showed significant differences from session to session, particularly in the negative SPAFF codes of Disgust and Low Domineering, and in the positive SPAFF codes of Interest, Affection, Humor, and Surprise/Joy. Interestingly for Aimee this change in client emotional expression—particularly with positive emotional expression—is positively associated with successful treatment outcomes (5).

### Parameters from mathematical models

The parameters generated from the math models are helpful for comparing sessions to each other using a common metric (6, 20, 21). Based on the results of the analysis, we can conclude that there is evidence for stability in each person’s parameters (particularly initial state and inertia) across the six sessions. While there may not have been significant differences between the sessions, one noteworthy comparison between the therapist and the client is that the initial state parameter for Jon Carlson are all positive, meaning that he started from a positive emotional state, while Aimee’s initial state was negative in four out of the six sessions and positive in two. Baker et al. (6) found similar results between the master therapists of three different approaches with all positive initial state parameters, while the clients started from negative initial states in half the sessions. This is consistent with the recommendations from the APA Task Force on Therapeutic Relationships against confrontations and negative processes (1), as well as (46) and Hill et al., (8) findings about therapist effects on clinical outcomes and clinical mastery.

At the same time, the inertia parameter (the tendency to stay in a previous emotional state; where lower scores are indicative of less inertia and higher scores are indicative of more inertia) had some dynamics worth exploring (20, 21). Jon Carlson’s inertia scores were generally higher than Aimee suggesting that in each session, he was less likely to move from one emotional state to another, and that Aimee was more likely to move from one state to another. One interpretation of this result was that Carlson remained more emotionally constant in the sessions, while Aimee was freer to move from her previous emotional responses throughout the session. This was corroborated by the significant differences in Aimee’s SPAFF scores, while Carlson’s did not change. Two notable exceptions were in the first and last session where Aimee’s level of inertia was greater than Carlson’s. In the first session, this might have been important because the therapist must make an effort to engage the client, and create the therapeutic relationship. While in the final session, Carlson was more expressive regarding the ending of therapy with Aimee, as evidenced by the fact that he was positive over 27% of the time, which was the largest percentage over the six sessions (see Table 1).

A final observation involves the parameters in session 3 and 4 (see Tables 3, 4), and the interaction between therapist and client. First, in session three, Carlson’s initial state was close to zero or neutral (0.019), while his inertia parameter was the highest of the six sessions (0.456). Aimee’s initial state was strongly negative for her (−0.413), while her inertial as also high (0.350). In this session, Carlson was actively pointing out Aimee’s ambivalence about confronting her mother’s behavior (which was a significant issue for Aimee), while Aimee was demonstrably uncomfortable about the issue (see Aimee’s comments below). In terms of the specific SPAFF codes, Carlson displayed the code Low Domineering (taking control of, or directing the conversation) 4.15% of the time, and the code Tension 2.74% of the time (which were the highest among the six sessions of each SPAFF code). For her part, Aimee demonstrated her discomfort by displaying the code Tension during 21% of the session, which was also the highest for her among the six sessions. While this may suggest the potential for a therapeutic rupture (38), in the next session, Aimee had the highest percentage of total positive SPAFF scores (nearly 12% of the time), and had her lowest level of inertia (0.118). Not surprisingly, this was after she was able to have a frank conversation with her mother about her sadness over the state of their relationship. Taken together, these results also highlight Carlson’s use of what he called using the “velvet hammer,” a skillful pattern of balancing therapeutic support and challenging (or positive and negative affect) during sessions (J. Carlson, personal communication, April 2, 2016). This is in keeping with several theories about the role and structure of the therapeutic relationship, including the Social Relations Model (39) or Wampold and Imel’s (2) Contextual Model. It is a good demonstration of how the affect coding and parameters derived from the mathematical model can provide a method to understand complex therapeutic interactions (6, 20, 21).

### Phase space portraits: Carlson and Aimee

Phase space portraits of Carlson’s six sessions with Aimee were created in order to explore the emotional dynamics between a master therapist and his client over a brief course of therapy (see Figure 1). In the first session, both client and therapist have trajectories toward an attractor in the therapist-positive/client-positive space (Figure 1A). This shows positive affect for both parties at the emotional endpoint for this session. In fact, Carlson acknowledges this:

I believe that my calmness… influences Aimee and she believes that I have seen this situation before… If Aimee feels a strong alliance with me, she will feel safe enough, and hopeful enough to want to talk about issues that previously were seen as too intimate, painful or taboo” [(26), p. 46].

However, in session 2 (Figure 1B), the attractor is in the client negative/therapist positive quadrant. This shift into mild client negativity may indicate an increase in therapeutic challenging and emotional heightening of the client for clinical purposes. Carlson as much as confirmed this idea when reviewing this session, saying: “Aimee’s negative self-talk has to be challenged and changed as it is very powerful and serves to limit what she can become” (p. 72).

In session 3 (Figure 1C), two possible attractors emerge within the portrait, one in the therapist-positive/client-negative space, and one in the therapist-positive/client-positive space. It seems that in this session, too much therapist positivity in response to more client negativity would result in greater client negativity. It is possible that excessive therapist positivity, when the client is expressing negative affect, may be interpreted as insensitive by the client. As Carlson reflected: “Aimee did a lot of work in this session. Her thinking and increased verbal participation showed her level of engagement. My role was to facilitate the discussion by asking questions and challenging her thinking in a positive manner” [(26), p. 125]. This is consistent with previous research indicating the critical role of therapist attunement to client emotions during sessions (40, 41) and demonstrating warmness, hopefulness, and empathy through the use of facilitative interpersonal skills (11).

In session 4 (Figure 1D), trajectories lead both client and therapist to an attractor in the positive/positive space, indicating a mildly positive emotional state for both parties at the end of this session. This shift toward positivity for both client and therapist at the end of session could represent a deliberate attempt by Carlson to augment his client’s positive affect as he approaches termination. In session 5, the attractor in the therapist-positive/client-negative space was only mildly negative for the client and mildly positive for the therapist (see Figure 1E). Finally, in session 6 (Figure 1F), Aimee ends the session in a mildly negative space while Carlson ends the session with mild positivity. While it may be surprising that Aimee ends this course of therapy with a mildly negative endpoint, during this session, client and therapist were discussing ways in which client has grown in therapy and how Aimee intends to carry this growth into her life after therapy with Carlson. Again, Carlson reflected on this session, stating:

In terminating therapy, I find it helpful to go over what has been accomplished and what is unfinished. It is also important to identify future areas of challenge where relapse might be more likely to occur and to obtain some commitment from the client to keep moving toward their goals [(26), p. 171].

In this context, Aimee’s somewhat negative affect at the end of this session may indicate her feelings about terminating the therapeutic relationship. Phase space portraits of these sessions conducted by Carlson may well depict his intentional management of both his own and his client’s emotional state (26). This finding is similar to (42) for therapist effects associated with treatment outcomes:

It is not about the therapist mimicking or offering a therapeutic relationship identically to the relationship desired or perceived by the patient but being open and flexible enough to recognize and accept the patient views on the bond and adapt his/her interactional style and therapeutic practice in accordance (p. 10).

Clearly, Carlson’s approach provided both the warmth that Aimee was seeking (as evidenced by the positive affect scores), but did not shy away from emphasizing the agency that she had to make changes in her life and relationships.

Ultimately, Aimee’s reflection over the course of her therapy, may be the most important indication of the overall success of the therapy. Sperry and Carlson (26) were able to ask Aimee for her feedback approximately 7 years following the course of therapy with Carlson. She said:

The aspect of our work together that stands out the most was the challenge to confront my mother. There was valuable time spent discussing my animosity toward her, but what seemed effective was the confrontation. I hesitate to use the word confrontation but that’s how I felt when I was faced with it. It was more of an overdue expression of feeling… The main changes that I made during our work together were to begin living my life with my mental health in mind. I needed to learn to be mindful of my needs instead of others’. Additionally, I needed to forgive my mother for the experiences I had in childhood. Once I began to forgive her, I felt as if I had begun to start healing from deeply embedded emotional needs wounds within me (p. 172).

Perhaps there is no better indication of a successful course of psychotherapy, than the testimony from a client almost 10 years after the termination of therapy. Aimee’s comment is consistent with findings from a Social Relations Model (and others) that patient reports of strong therapeutic relationships were directly linked with better outcomes (42). Both Carlson and Aimee credit the relationship that they built in the six sessions as being an important factor in the success of the therapy.

## Limitations and future directions

The present study makes a unique contribution to the literature on in-session affect, therapeutic expertise, and in-session client and therapist indicators in the development of an effective therapeutic relationship. While most studies of therapist affect look at therapist and client affect as measured before and after session, this study examined the within-session affective dynamics of therapy. Perhaps the greatest value of this study is the way in which it addresses a significant gap in the research on both therapeutic expertise and the affective dynamics of effective therapy (1, 6, 8, 10, 11).

The present study was not without some limitations. As Hill et al. (8) noted, many studies of expertise lack statistical power due to low numbers. This project was focused on one course of therapy that was relatively brief (six sessions). Investigating longer-term therapeutic relationships may yield deeper insights into how these relationships are developed and change over time. While the results of this study was similar to another investigation of expert therapists using DS modeling (6), there are other avenues to pursue including how this investigation of therapeutic relationships (using observer coding) differ depending on client and therapist self-report measures of the therapeutic alliance (8, 11). In addition, other modeling procedures (e.g., Bayesian modeling), may provide additional insights to the emotional exchange between therapists an dlcients. Lastly, while both participants rated the course of therapy to be successful, there were no contemporaneous measures of outcome or symptom reduction reported. Future research using DS modeling with expert or non-expert clinicians would benefit from including outcome measures.

Another limitation of the coding system used is the significant percentage of time that is coded Neutral. This stems from the definition of the code (when one person is talking, unless there is convincing evidence of additional affect codes present, the code is Neutral), the fact that as one person is talking, the other person is listening (particularly in therapy), or when there is a segment of time that is uncodeable, then Neutral is the default code. There may be more subtle affect displays that are not well-accounted for in SPAFF that may be detected using computer vision, affective computing and machine learning which may facilitate more real-time feedback to clinicians (5, 6, 43, 44).

## Conclusion

The purpose of this paper was to use DS modeling of observational measures of emotional expression between therapists and clients in a complete course of psychotherapy to determine the stability as well as the changes in the therapeutic relationship observed over different sessions. Overall, the results yielded some important implications for future research as well as clinical practice and training. Carlson’s ability to stay emotionally positive and relatively stable across the six sessions (relative to Aimee) is noteworthy. It formed the basis for a stable base from which she could explore alternative methods to relate to others that she had allowed to dictate her actions (i.e., her mother, father, and ex-husband). This is in keeping with previous research on the role of therapist facilitation of the therapeutic relationship, emotional expression within the therapeutic relationship, and influence of these on client outcomes (5, 7, 8, 11, 27, 42). Investigating expert psychotherapists using dynamical systems mathematical models is a useful approach for understanding complex phenomena in psychotherapy. Further research is needed into the use of therapist affect to develop high-quality therapeutic relationships, assess and repair therapeutic ruptures, and monitor client affect for indications of clinical progress and relationship strength.

## Clinical significance/Impact statement

Dynamical systems mathematical modeling can be used to explore the complexities of the therapeutic relationship. Such an analysis allows for complex study and prediction of outcome based on the coding of interpersonal affective behaviors of video-recorded psychotherapy as it unfolds over time. It can be used as an instructional and exploratory tool to explore the process of creating change with clients in psychotherapy.

## Data availability statement

The raw data supporting the conclusions of this article will be made available by the authors, without undue reservation.

## Ethics statement

The studies involving human participants were reviewed and approved by Florida Atlantic University. Written informed consent for participation was not required for this study in accordance with the national legislation and the institutional requirements. Written informed consent was obtained from the individual (s) for the publication of any potentially identifiable images or data included in this article.

## Author contributions

PD, PP, RF, AB, and GP contributed to the design, coding, data collection, and analysis and reporting of this article. All authors contributed to the article and approved the submitted version.

## Conflict of interest

The authors declare that the research was conducted in the absence of any commercial or financial relationships that could be construed as a potential conflict of interest.

## Publisher’s note

All claims expressed in this article are solely those of the authors and do not necessarily represent those of their affiliated organizations, or those of the publisher, the editors and the reviewers. Any product that may be evaluated in this article, or claim that may be made by its manufacturer, is not guaranteed or endorsed by the publisher.

## Footnotes

^1^Due to space limitations, in-depth discussions of SPAFF coding procedure, as well as additional information regarding SPAFF (e.g., definitions) please consult the Supplementary material for this article.

^2^More information on the training procedure is included in the Supplementary material.

^3^The coder operates a keyboard with codes assigned to different keys. When a code is detected, the coder presses the appropriate key for the code. Each second is assigned the code until the coder presses another key for another code. As a result, a continuous second-by-second data stream is created. See supplemental material for more information.

^4^For a more in-depth explanation of the equations, parameters, and the modeling procedure, please see Baker et al. (6), as well as the Supplementary material for this article.

^5^These two parameters are combined to created uninfluenced steady state combining the inertia parameter (resistance to change), and the initial state (the individual’s dispositional characteristics) using the formula: b_1_/m.

^6^For specific information on the derivation of parameters, please see Gottman et al. (17).

^7^The uninfluenced steady state is a ratio of b/m parameters, see Gottman et al. (17).

^8^Several programs are available to accomplish this, such as the ‘dyad’ package written in the R programming language, and freely available (47).

^9^Although any ODE solver in R or Python would be just as useful.

^10^Again, a more thorough discussion of these topics are in the Supplementary material.

^11^The phase portraits can also be rendered in a three-dimensional phase-space portraits of the dynamic nonlinear models using time steps as the z-axis. These are available upon request.

## Supplementary material

SUPPLEMENTARY FIGURE S1 Influence Functions and Phase-Space Portrait for Generic Model.

SUPPLEMENTARY FIGURE S3 Time Series scatterplots of SPAFF scores for all six sessions of therapy between Jon Carlson and Aime. **(A)** is the phase portrait for session 1, **(B)** is for session 2, **(C)** is for session 3, etc. Colors denoted by the bar on the right signify the time (in seconds) in the session for the data point.
