# Supplementary material for: Understanding the role of emotion and expertise in psychotherapy: An application of dynamical systems mathematical modeling to an entire course of therapy
Source: Front Psychiatry. 2023 Apr 11;14:980739. doi: 10.3389/fpsyt.2023.980739 (PMC10126830; doi:10.3389/fpsyt.2023.980739)
Supplement: Supplementary file 1 [file Data_Sheet_1.docx]

Supplemental Material

The Specific Affect Coding System (SPAFF)

The Specific Affect Coding System, or the SPAFF, was originally developed (Gottman & Krokoff, 1989) and validated (Krokoff & Gottman, 1989) for the examination of the marital relationship. This system was created based on an earlier system called the Couples Interaction Scoring System (CISS, Gottman, 1979) and the Facial Action Coding System (Ekman & Friesen, 1978). This system began as a ten code system, but was later expanded to include twenty behavioral codes (Coan & Gottman, 2007). In the twenty code system, there is one affect code for neutral behavior, seven positive affect codes (affection, high validation, humor, interest, surprise/joy, low validation and tense humor), and twelve negative affect codes (contempt, belligerence, criticism, stonewalling, defensiveness, high domineering, low domineering, anger, sadness, whining, disgust, and tension) (Gottman et al., 1998). These codes are applied in real-time via marked keys on a keyboard while watching video-taped interactions uploaded into observational research software. This creates a second-by-second data stream of the interaction.

As indicated by Heyman (2001), “Because the SPAFF was the first second-generation coding system developed, it has by far the best evidence of construct and criterion validity for its constructs.” (p. 25). It is indicated that the codes for affection, anger, belligerence, contempt, domineering, humor, sadness, and validation have discriminant validity as well (Heyman, 2001). Further, SPAFF has been shown to have predictive validity as it has reliably predicted divorce (Gottman & Levenson, 1992; Gottman & Notarius, 2000). It has also been shown to have concurrent validity with the Rapid Couples Interaction Coding System (a later iteration of the CISS, the precursor to SPAFF) (Coan & Gottman, 2007; Gottman et al., 2002).

**Video affect coding procedure.** The therapists’ and clients’ affective exchanges from the *Psychotherapy over Time* (Carlson, 2006) videos were coded using the twenty code SPAFF (Gottman et al., 1998). The twenty code manual provided by the Gottman Institute (Gottman et al., 1998) was the basis for training the coders employed by the current study. All coders employed in the current study were involved in intensive three-day (eight hours per day), SPAFF coding training conducted by a Gottman Institute coding trainer. The coders then coded ten videos over the course of the next few weeks, and maintained inter-rater reliability with the Gottman Institute coding trainer (gold standard coder) for the last six consecutive videos, as per the procedure established by the Gottman Institute for coders (K. Drummey, personal communication, November, 10, 2012). Coders are considered to have achieved inter-rater reliability in SPAFF coding if their codes show windowed kappa, free-marginal kappa, and windowed free-marginal kappa coefficients of .6 or higher (K. Drummey, personal communication, November, 10, 2012). Due to SPAFF being a real-time coding system, the procedure for attaining inter-rater reliability differs from what may typically be expected, and the expected kappa coefficients are lower to account for the nature of the way the material is coded (J. Gottman, personal communication, February, 1, 2016).

Three psychotherapy sessions for each of the two videos (six sessions in total) were separated into fifteen minute video segments using a video editing program, iMovie 11 for iOS. These segments were then uploaded into The Noldus Observer XT 11 (observational coding software) to be coded using SPAFF. The sessions will be SPAFF coded in segments of fifteen minutes to prevent coder fatigue, as each segment requires three views to be coded. The first viewing is to establish a contextual baseline, the second viewing is to code one of the parties (i.e., the therapist), and the third viewing is to code the other party (i.e., the client). The coded data is then exported for manipulation in statistical programs. This procedure was established in Gottman’s research during the validation of the SPAFF model (Coan & Gottman, 2007), and has since been replicated to its use in research on the therapeutic relationship (Luedke et al., 2017).

For the mathematical modeling, each second of material was assigned a code and each code was weighted; then every six seconds of material was summed to create 150 data points from 900 seconds (fifteen minutes) of video (Gottman et al., 2002; Luedke et al., 2017). Gottman et al. (2002) created these “interactive units” (p. 340) in previous research to allow for more variability in the data, which also allows for creating dynamic nonlinear mathematical models. The weighting of the codes varies from -4 to +4 so a range of -24 to +24 for each unit is possible (for original weighting see, Gottman et al., 2002). This weighting range has been translated to research on other relationships, although the codes have been re-weighted to fit the therapeutic relationship (Luedke et al., 2017). Luedke et al. (2017) posited that the therapeutic relationship carries an inherent power differential that is not assumed in a marital relationship, and completed a discriminant function analysis on a sample of SPAFF coded therapeutic interactions to create appropriate weights for codes that differ for clients and therapists. This updated weighting system was employed in the current study. The current study used Excel by Microsoft, Statistical Package for the Social Sciences by IBM, and MATLAB by MathWorks to process raw data based on previously established procedures (Luedke et al., 2017).

SPAFF Definitions**.** An objective, holistic, and hierarchical affect coding system originated in 1989 by Gottman & Krokoff (Coan & Gottman, 2007), that was later expanded to a 20 code version (Gottman et al., 1998) which is employed in the current study. **“**It draws on facial expression (based on Ekman and Friesen’s Facial Action Coding System; Ekman and Friesen, 1978), vocal tone, and speech content to characterize the emotions displayed” (Gottman, Murray, Swanson, Tyson, & Swanson, 2002, p. 339). For in-depth and complete definitions for codes see Gottman et al. (1998) and Coan and Gottman (2007).

**Positive affects (positive SPAFF codes).** Affection, high validation, humor, interest, surprise/joy, low validation, and tense humor (Gottman et al., 1998).

**Affection.** Direct expressions of empathy, connectedness, tenderness, or compliments which are often accompanied by mirroring the affect of the other person (Gottman et al., 1998).

**High validation.** Showing acceptance or understanding of another’s perspective through paraphrasing, finishing sentences, or apologizing (Gottman et al., 1998)

**Humor.** Shared moments of laughter and feelings of happiness during giggling, mutual jokes, and good natured teasing (Gottman et al., 1998).

**Interest.** Open-ended or follow-up questions in which someone is seeking further information, thoughts, or emotions (Gottman et al., 1998).

**Surprise/joy.** A reactionary response of happiness or positive feelings which is detectable in facial affect by clearly defined smiles and widened eyes. A “positive, happy or emphatic reaction to some event” (Gottman et al., 1998, p.24).

**Low validation.** Positive backchannels including head nods and other forms of verbal/ non-verbal acknowledgement of a person sharing (Gottman et al., 1998).

**Tense humor.** Brief moments of shared humor or laughter that is often accompanied by nervous or tense affects. The laughter or shared humor typically ends abruptly (Gottman et al., 1998).

**Negative affects (negative SPAFF codes).** Contempt, belligerence, criticism, stonewalling, defensiveness, high domineering, low domineering, anger, sadness, whining, disgust, and tension (Gottman et al., 1998).

**Contempt.** An attempt to insult or demonstrate lack of respect toward another often through sarcasm, mockery, or insults; and/or accompanied by discrete facial affect shown to be associated with contempt universally (Gottman et al., 1998).

**Belligerence.** Purposeful or intentional behavior in which a person shows intent of provoking a fight or negative response (Gottman et al., 1998).

**Criticism.** Characterological attacks, blaming, listing complaints, or complaining using absolute language directed at another person (Gottman et al., 1998).

**Stonewalling.** Discontinuing response with no vocal acknowledgement or backchannels, and very little or no eye contact often accompanied by emotional flooding (Gottman et al., 1998).

**Defensiveness.** Making excuses, counter-criticism, and showing verbal blamelessness in response to perceived or felt criticism (Gottman et al., 1998)

**High domineering.** Attempting to control another’s behavior or response through emotional or physical threats (Gottman et al., 1998).

**Low domineering.** Attempting to control the conversation or force compliance through incessant speech or interruption (Gottman et al., 1998).

**Anger.** A reactionary response of anger, frustration, or annoyance which is detectable in facial affects, tone, expression, and gestures (Gottman et al., 1998).

**Sadness.** A reactionary response of sad affect; “decrease in energy and a passive, resigned countenance” (Gottman et al., 1998, p. 17).

**Whining.** A complaint stated with a vocal tone which is higher-than-baseline, nasally, or sing-song in quality (Gottman et al., 1998).

**Disgust.** A reactionary response “verbal and non-verbal rejection of some kind of noxious stimulus” (Gottman et al., 1998, p. 4).

**Tension.** Fidgeting, shifting, verbal pauses, and nervous laughter often accompanied by an uncomfortable feeling (Gottman et al., 1998).

**Neutral affects (neutral SPAFF codes).** Neutral (Gottman et al., 1998).

**Neutral.** Any interaction which does not fit other behavioral categories typically represented by matter of fact exchanges without other verbal or non-verbal cues indicating other affects. Non-codable interaction representing the division between positive and negative codes (Gottman et al., 1998).

***Video affect coding procedure***

The therapists’ and clients’ affective exchanges from the *Three Approaches* (Brown & Tullos, 2012a, 2012b) videos were coded using the twenty-code SPAFF^^[[1]](#footnote-1)^^ (Gottman et al., 1998). The twenty-code manual provided by the Gottman Institute (Gottman et al., 1998) was the basis for training the coders employed by the current study. All coders employed in the current study were involved in intensive three-day (eight hours per day) SPAFF coding training conducted by a Gottman Institute coding trainer. The coders then coded ten videos over the course of the next few weeks, and maintained inter-rater reliability with the Gottman Institute coding trainer (gold standard coder) for the last six consecutive videos, as per the procedure established by the Gottman Institute for coders (K. Drummey, personal communication, November, 10, 2012). Coders are considered to have achieved inter-rater reliability in SPAFF coding if their codes consistently show windowed kappa, free-marginal kappa, and windowed free-marginal kappa coefficients between 0.6 and 0.7 or higher (K. Drummey, personal communication, November, 10, 2012). Due to SPAFF being a real-time coding system, the procedure for attaining inter-rater reliability differs from what may typically be expected, and the expected kappa coefficients are lower to account for the nature of the way the material is coded (J. Gottman, personal communication, February, 1, 2016).

**Mathematical Modeling**

Gottman and his colleagues originally designed an approach to assess the relationship dynamics (particularly emotional exchanges) between spouses using dynamical systems nonlinear equations (see Gottman et al., 2002 for details). Liebovitch et al. (2011) modified these equations, and Peluso et al. (2012) created simulations of different relationship dynamics by manipulating the parameters of the mathematical model. Peluso et al. (2018), using a limited data set, demonstrated that data on emotional expressions gathered by observational coding of the therapeutic relationship could be analyzed using a dynamical systems mathematical model. In this section, we will describe the equations and parameters in detail that will be modelled in the present analysis, as well as the influence functions, phase-portraits, and a description of the repair function that will be used in the models.

**Math Modelling Equations and Parameters.** Gottman and his associates (Cook et al., 1995; Gottman et al., 2002) presented their equations to demonstrate how the emotional state of one partner (e.g., wife) at a particular time point (*W_t+1_*) is a function of their emotional state one time point previous (*W_t_*), their inertia to change (*r*), their initial state (*a*)—which is a constant— and the influence of a partner (e.g. husband) at the same time point (*I_hw_(H_t_)*). A reciprocal equation for the other partner is likewise written.


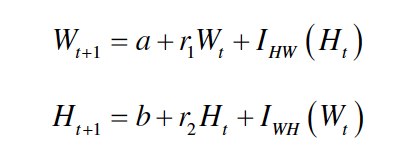


As a result, the system is dynamic (reacting differently based on the individual’s previous emotional state, resistance to change that emotional state, and the influence of one person on the other), and closed (only the two actors involved).

Liebovitch et al. (2011), used the differential form of the equation to model the relationship dynamics of therapists (T) and clients (C). According to Liebovtich et al. (2008), the benefit of these equations:

We study the differential form of these equations because doing so allows us to use standard software for numerical integration, and because it makes available to us powerful theorems and analytical methods that are only applicable to continuous (and not discrete) systems, so that we can more completely derive the dynamics. Perhaps this is also even more realistic as it represents a continuous interaction between the actors rather than alternating discrete equally timed events represented by the discrete model. (p. 6375).

Specifically this form of the equations calculates the changes in therapists’ score (*dT*) or the change in the clients’ score (*dC*) over time (*dt*).


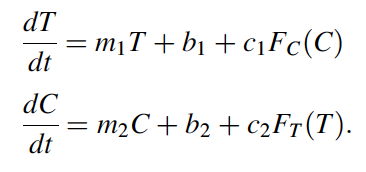


The equation uses the same parameters listed above: initial state (*b_1_* and *b_2_* are the same as *a* and *b*), and inertia (*m_1_* = 1- *r_1_*, and *m_2_* = 1- *r_2_*) (Leibovitch et al., 2008, 2011; Peluso et al., 2012).*_._* The influence functions of the therapist on the client (*c_1_F_T_(T)*) and of the client on the therapist (*c_1_F_C_(C)*) will be detailed below.

**Influence Functions and Phase Portraits.** The influence function is a mathematical representation of the relative strength of the impact of one partner’s emotional behavior on another partner at any given point in time, based on the level of positivity or negativity of the other partner. Gottman and his associates (Gottman et al., 2002) knew from their empirical studies that this influence was not constant, but was dependent on whether the other partner was positive or negative. According to Gottman and Peluso (2018), “negative affect at time *t* was more powerful than positive affect in predicting the partner’s next behavior at time t + 1. This was assessed by a steeper slope for negative than for positive affect in the influence functions” (p. 20). Thus, these multi-linear influence functions, with different slopes for different regions of affect, allowed them to quantify these changes more accurately.

***Influence Functions*.** When creating the influence function for therapists on clients (*F_T_(T)*) and clients on therapists (*F_C_(C)*), Leibovitch et al. chose a three linear segment influence. Specifically, the linear segments add different strengths (or weight) and have different slopes based on either the client or therapist’s affect. The first element in the dynamical system is the influence function (see Figure S1a). The generic tri-linear influence function in Figure S1a, where the therapist’s influence on the client (*F_T_(T)*) is depicted in blue, while the client’s influence on the therapist (*F_C_(C)*) is depicted in red is a generic depiction of the relative influence of both parties to one another (please see Gottman et al., 2002; Liebovitch et al., 2011 and Peluso et al., 2012, for a thorough discussion of these).

For the therapist’s influence on the client ((*F_T_(T)*)), these linear segments were written mathematically as follows:


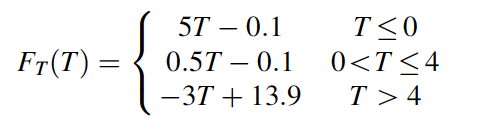


In more real-world terms, when the therapist is neutral (T=0), the client is likely to be neutral, and as the therapist begins to get negative (T$\leq0)$, the client is likely to be sensitive to this and become negative as well (even more steeply). Lastly, as the therapist becomes more positive in affect (T > 0), the client will match the positivity, but more gradually than the way that the therapist attempts to match the client (above). But, as with the therapist, this is only up to a point, when the client will turn negative in the presence of extreme positive affect (though much more steeply than the therapist does above).

Recently, Baker et al. (2022) determined that the original threshold points and slope for the influence function for clients on therapists (*F_C_(C)*), did not adequately model the relationship. As a result, a slight modification from Peluso et al. (2012) was made:

*
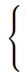
*

*C* + 2 *C* <= 0

*F_C_(C) =* 0.4*C +* 2 0 *< C <* 2.5

-1.4*C*+ 6.5 *C* > 2.5

Thus, when the client is neutral (C=0), the therapist is slightly more positive, and as the client begins to get negative (C$\leq0)$, the therapist will attempt to stay positive. Finally, as the client becomes more positive in affect (C > 0), the therapist will match the positivity, up to a point, when they will then start to turn negative (in the face of extreme, or manic, positive affect).

The influence functions rely on the threshold parameters (positive and negative) to determine where the linear segments change to create three regimes (broadly speaking, negative, neutral and positive). In the (F_T_(T)) equation, the threshold between negative and neutral is at T=0, while the threshold between neutral and positive is at T=4. For the F_C_(C)) equation, the threshold between negative and neutral is at C=0, while the threshold between neutral and positive is at C=2.5.

Another important feature of dynamical systems models are attractor points, repulsion points or a saddle nodes. These occur in a dynamical systems model where the two influence functions intersect, often referred to as nullclines, or critical points, in the system. A nullcline is a point in which the change of the affective emotional valence of the client or therapist equals zero (Liebovitch et al., 2011; Peluso et al., 2018).

***Influence Function Graphs.*** Phase portraits created by inputting the derived parameters: inertia, initial state, the influence functions for therapist and client, and the repair function (see below) into the differential equations (detailed above) using an ODE solver and graphed in MATLAB (again, see Liebovitch et al., 2011; Peluso et al., 2018 for details). The phase portrait allows for a more complete exploration of the system by creating trajectories from each potential starting point coordinates (see Figure S1b). As mentioned above, critical points, or steady states, in the system exist where nullclines intersect and serve as important indicators of the system dynamics. Graphically, it is where the influence function of one person intersects with the influence function of the other person (Liebovitch et al., 2008). The critical points either are attractor points that pull the dynamic towards the attractor (represented as a black dot in the upper right hand, or positive-positive, quadrant in Figure S1b), a repulsion point (that directs the dynamic away from a point (not depicted in Figure S1b), or a saddle node (where there is neither attraction of repulsion—shown in the negative-negative quadrant in Figure S1b). These models have previously been represented using data from simulated psychotherapy (see Peluso et al., 2012; Peluso et al. 2018).

***Influence Functions and Repair Terms for All Six Sessions.***

Figure S.2 displays the influence functions of all six sessions, with the first 3 sessions in the top row (Figures S.2a-c), and the last 3 sessions on the bottom row (Figures S.2d-f). In looking at the phase portraits in the manuscript (Figure 1 of the manuscript), the complex nature of the figures sometimes obscures some of the underlying dynamics of each individual session. However, in considering the Influence Functions in Figure S.2 each of the unique dynamics that occurred in each session. These are all based on the positive and negative threshold parameters listed in Tables 3 for Jon Calrson and Table 4 for Amiee (the client) in the manuscript, which were derived from the SPAFF coding. At first glance of the parameters for Jon Carlson in Table 3, the uninfluenced steady state was positive (this is a ratio of the initial state and inertia parameters), while the uninfluenced steady state for Amiee (in Table 4), 4 out of the 6 sessions were negative. The parameters for the influence functions (positive and negative threshold) were fairly consistent across all six sessions, with the negative threshold between -0.5 and -2.7, while the positive thresholds ranged from -1.6 to 0.9 for the Jon Carlson, while for the Amiee’s thresholds there was more variability ranging from -2.6 to 1.4 and -0.2 to 6.2 for the negative and positive thresholds, respectively.

The combination of the positive and negative threshold parameters are best visualized in Figure S.2. For example, in session 3 (Figure S.2c) both of Carlson’s thresholds (between negative and neutral) is -3 and the positive threshold is -1.3 (both in the positive-positive quadrant, depicted by the red line), at the same time Amiee’s negative threshold is -1.2 and her positive threshold is 3.4 (depicted by the blue line). This places both of Carlson’s thresholds in the therapist positive-client negative quadrant. This does not mean that all of the nullclines are in any one quadrant, as one can plainly see that there are 2 nullclines in the therapist positive-client negative quadrant (one becomes a saddle node in the phase space portrait in Figure 1, while the other becomes an attractor), and there is an attractor in the client positive-therapist negative quadrant. What is most noteworthy is how the locations of the threshold parameters changes the influence functions, so while they may be based on Peluso et al.’s (2012) generic model (Figure 1), when parameters derived from actual session data is used (including the initial and inertia parameters), the results can be radically different.

Next, the impact of the repair function on the influence function will be considered. Overall, the strength of Jon Carlson’s repairs ranged from 0.4 to 4.1 in 5 of the 6 sessions, while clients repair strength ranged from 1 to 3.1 also in 5 of the 6 sessions. Graphically, this may be best exemplified looking at 2 separate graphs. Again, looking at Jon Carlson’s influence function with Amiee in the first session (Figure S.2a), there is a strong repair (4.1) that begins when the client is at -2.6. This eliminated a nullcline that would have occurred in the therapist positive-client negative quadrant, as well as another nullcline in the negative-negative quadrant (which is indicative of a poor therapeutic relationship)^^[[2]](#footnote-2)^^. Possibly the most dramatic example of the impact of the repair function is in the third session (Figure S.2c). Carlson initiates a moderate repair (1.7) when the client score hits -3.7, The consequence of this is that an attractor point was *not* created in the negative-negative quadrant (as would have been created without the repair function), or would have been created if the repair was weaker. At the same time, Amiee also had a moderately strong repair (1.3) when Carlson’s score reached -1. Recently, Eubanks et al. (2021) have asserted that repairing ruptures represents a transtheoretical element of evidence-based practice, and that responsive repairs are critical for clinical success. These models of an acknowledged master in the area of psychotherapy seems to be in alignment with this statement.

**Comparison of Model Graphs with Ground Truth Graphs**

Dynamical systems models are mathematical, but not necessarily statistical (Strawinska-Zanko & Liebovitch, 2018). As a result, one of only ways to discern whether a model (or its parameters) accurately models what happened within the original system, and/or is valuable for making predictions, is to compare it to the actual events that occurred (often called “ground truth”) (Baker et al., 2022; Fernandez & Picard, 2011; Madhyastha et al., 2011). For the six models presented here, we have taken the original data set used to generate the parameters, and plotted them as a scatterplot time series. Similar to Figure 1 in the article, Figure S3 plots Carlson and Amiee’s weighted SPAFF score (which was used to generate the parameters for the model), as a scatterplot over time (depicted by the color coding gradient^^[[3]](#footnote-3)^^ on the right side). When comparing the two figures, one of the first similarities is how much time is spent in the quadrants, and how it corresponds to the attractor in the quadrant. For example, in Figure 1a (session 1), there are two attractor points (one in the therapist positive/client negative quadrant, one in the therapist/client positive quadrant). In Figure S3a, those same 2 quadrants are where the majority of time was spent in the session. In addition, the two sets of graphs seem to align when comparing the final states of the sessions in Figure 1 with the final data points in Figure S3. In each of the six sessions, the end of the session in Figure S3 seems to echo where the attractor is in the corresponding phase space portrait in Figure 1. It is important to remember that the phase-space portraits represent all the possible combinations of starting points for client and therapist, whereas the scatterplot represents only one of these potential pathways. Taken together, the results of these analyses provide some interesting insights into the dynamics of Carlson’s and Amiee’s relationship and how it evolved over time. While the results of the individual SPAFF codes reveal differences in the expressed affect of Amiee, Carlson’s codes remained relatively consistent. Furthermore, the graphs in Figures 1 & S3 show how these parameters come together to represent the affective exchange in the therapeutic relationship as a dynamic system.

**References**

Baker, A., Peluso, P.R., Freund, R., Diaz, P. & Ghaness, A. (2022). Using Dynamical Systems Mathematical Modelling to Examine the Impact Emotional Expression on the Therapeutic Relationship: A Demonstration Across Three Psychotherapeutic Theoretical Approaches. *Psychotherapy Research, 32*(2), 223-237. DOI: 10.1080/10503307.2021.1921303

Coan, J. A., & Gottman, J. M. (2007). Specific affect coding system. In J. A. Coan &J. J. B. Allen (Eds.),*Handbook of emotion elicitation and assessment* (pp. 106-123). New York, NY: Oxford University Press

Ekman, P., & Friesen, W. V. (1978). *Manual for the facial action coding system*. Palo Alto, CA: Consulting Psychologists Press.

Gottman, J. M. (1979). Detecting cyclicity in social interaction. Psychological Bulletin, 86(2), 338–348. [https://doi.org/10.1037/0033-2909.86.2.338](https://psycnet.apa.org/doi/10.1037/0033-2909.86.2.338)

Gottman, J. M., & Krokoff, L. J. (1989). Marital interaction and satisfaction: A longitudinal view. *Journal of Consulting and Clinical Psychology, 57*(1), 47-52.doi:10.1037/0022-006X.57.1.4

Gottman, J. M., & Levenson, R. W. (1992). Marital processes predictive of later dissolution: Behavior, physiology, and health. Journal of Personality and Social Psychology, 63(2), 221–233. [https://doi.org/10.1037/0022-3514.63.2.221](https://psycnet.apa.org/doi/10.1037/0022-3514.63.2.221)

Gottman, J., Murray, J., Swanson, C., Tyson, R., & Swanson, K. (2002). *The Mathematics of marriage: Dynamic nonlinear models.* Cambridge, MA: MIT Press.

Gottman, J. M., & Notarius, C. I. (2000). Decade review: Observing marital interaction. Journal of Marriage and the Family, 62(4), 927–947. [https://doi.org/10.1111/j.1741-3737.2000.00927.x](https://psycnet.apa.org/doi/10.1111/j.1741-3737.2000.00927.x)

Gottman, J. M., & Peluso, P. R. (2018). Mathematics of Marriage. In U. Strawinska-Zanko & L. Liebovitch (Eds.) Mathematical Modeling of Social Relationships - What Mathematics Can Tell Us About People*.* (pp.17-30)*.* New York, NY: Springer

Gottman, J. M., Woodin, E. M., Coan, J. A. (1998). *Specific Affect Coding Manual 20 Code version (4.0)*. Unpublished manuscript

Heyman, R. E. (2001). Observation of couple conflicts: Clinical assessment applications, stubborn truths, and shaky foundations. *Psychological Assessment, 13*(1), 5–35. [https://doi.org/10.1037/1040-3590.13.1.5](https://psycnet.apa.org/doi/10.1037/1040-3590.13.1.5)

Liebovitch, L. S., Peluso, P. R., Norman, M. D., Su, J. & Gottman, J. M. (2011). Mathematical Model of the Dynamics of Psychotherapy. *Cognitive Neurodynamics.* doi: 10.1007/s11571-011-9157-x

Leudke, A., Peluso, P.R., Diaz, P., Baker. A., & Freund, R. (2017). An analysis of the first session: Measuring affect in the therapeutic relationship to predict dropout and return. *Journal of Counseling and Development, 95*(2), 125-135*.*

Peluso, P. R., Liebovitch, L. S. Gottman, J.M., Norman, M. D. & Su, J. (2012). A Mathematical Model of Psychotherapy: An Investigation Using Dynamic Non-linear Equations to Model the Therapeutic Relationship. *Psychotherapy Research, 22* (1), 40-55. doi: 10.1080/10503307.2011.622314

Peluso, P. R., Diaz, P., & Taibi, G. (2018). Quantitative Video Coding of Therapist-Client Sessions. In U. Strawinska-Zanko & L. Liebovitch (Eds.) *Mathematical Modeling of Social Relationships - What Mathematics Can Tell Us About People.* (pp.31-50)*.* New York, NY: Springer

1. The method of coding typical to the SPAFF was also altered due to the camera angles being different than typical procedure when sessions are recorded by researchers. Because of the pre-recorded nature of the film series, researchers adjusted for implied process in the non-visible participant, and the results of these adjustments cannot be known. Upon viewing examples of this issue within the videos, John Gottman agreed with the proposed adjustments and did not think the limitation would create meaningful differences in the results (J. Gottman, personal communication, May, 23, 2017). [↑](#footnote-ref-1)
2. This also created more opportunities in the therapist positive-client negative quadrant for trajectories to go to the attractor in the positive-positive quadrant as well. [↑](#footnote-ref-2)
3. Color graphs are available in the online version of this article [↑](#footnote-ref-3)
