# Supplementary figures and images for: Understanding the role of emotion and expertise in psychotherapy: An application of dynamical systems mathematical modeling to an entire course of therapy
Source: Front Psychiatry. 2023 Apr 11;14:980739. doi: 10.3389/fpsyt.2023.980739 (PMC10126830; doi:10.3389/fpsyt.2023.980739)

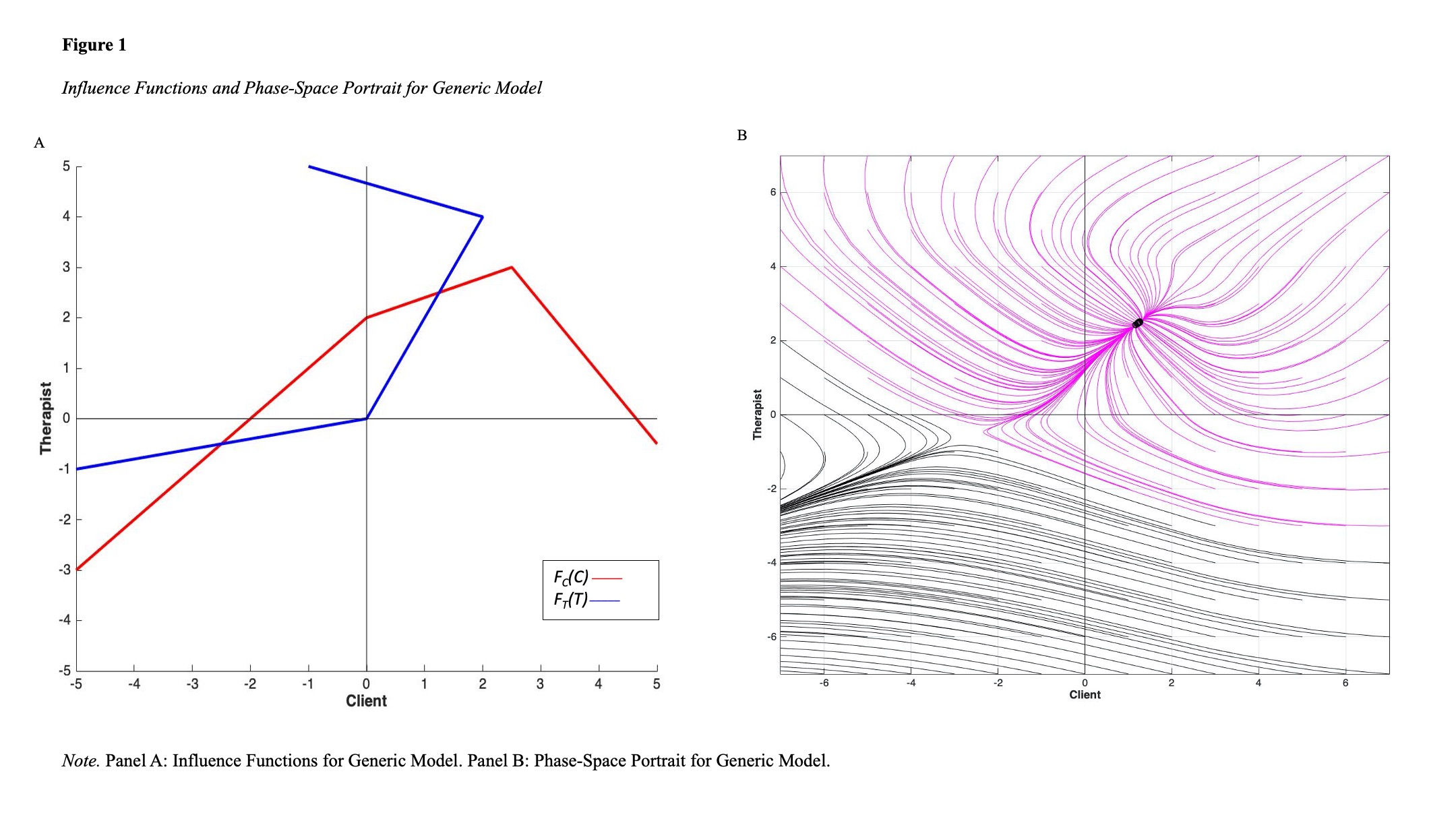

Supplement: SUPPLEMENTARY FIGURE S1 — Influence Functions and Phase-Space Portrait for Generic Model. [file Image_1.JPEG]

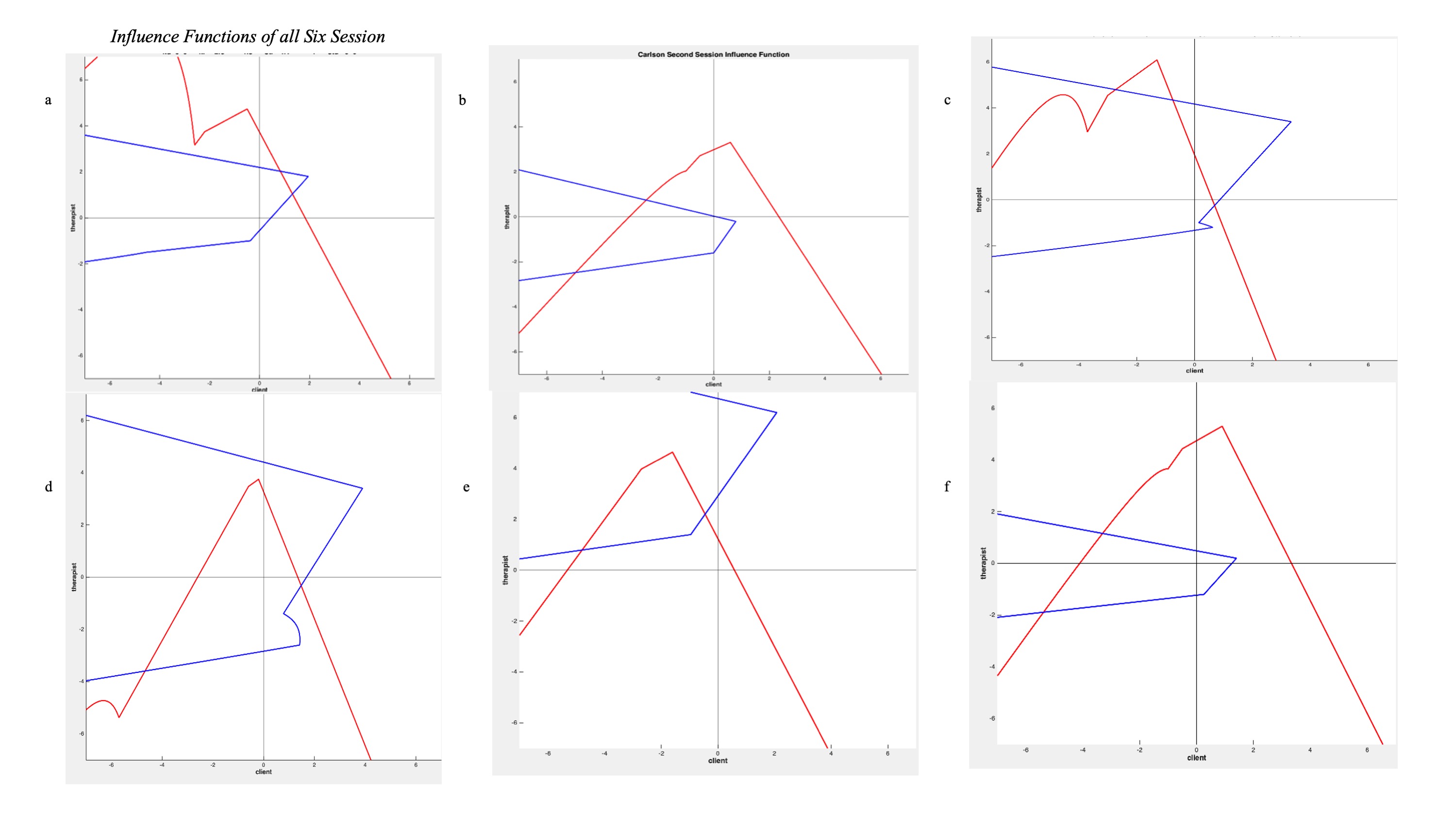

Supplement: Supplementary file 3 [file Image_2.JPEG]

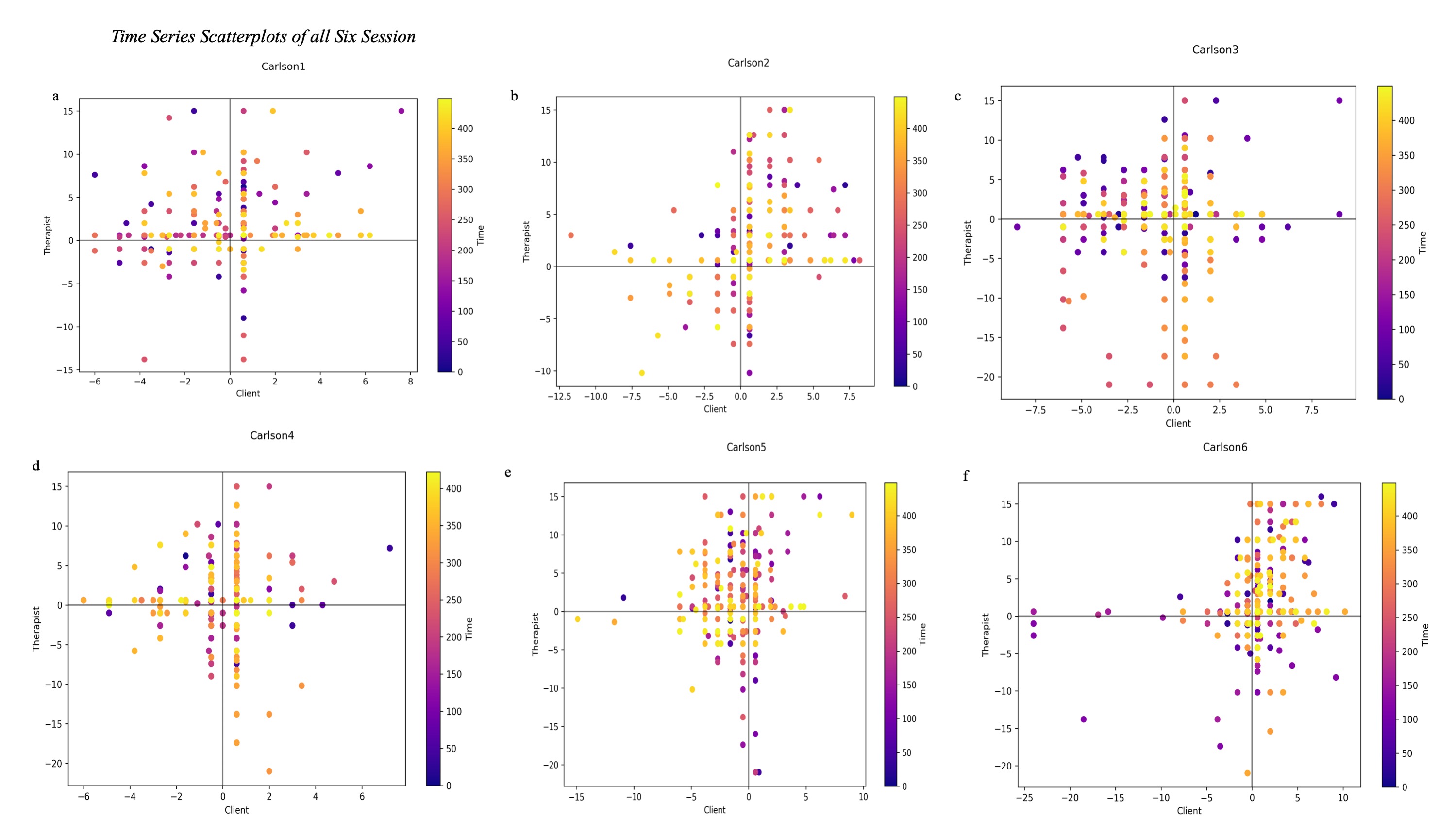

Supplement: SUPPLEMENTARY FIGURE S3 — Time Series scatterplots of SPAFF scores for all six sessions of therapy between Jon Carlson and Aime. (A) is the phase portrait for session 1, (B) is for session 2, (C) is for session 3, etc. Colors denoted by the bar on the right signify the time (in seconds) in the session for the data point. [file Image_3.jpg]
